# Protocol to evaluate human thermoregulation before and after thermal stress

**DOI:** 10.1016/j.mex.2024.102977

**Published:** 2024-10-02

**Authors:** Mariluz Castrillón-Gutiérrez, Natali Olaya-Mira, Carolina Viloria-Barragán, Julieta Henao-Pérez, Edison Alejandro Álvarez -David, Gloria Díaz-Londoño

**Affiliations:** a Centro de Ayudas Diagnósticas Cardiovasculares-Cardioestudio, Medellín, Colombia; b Biomedical Research and Innovation Group (GI2B), Faculty of Exact and Applied Sciences, Metropolitan Technological Institute, Medellín, Colombia; c Cooperative University of Colombia, Faculty of Health Sciences, Medicine, Medellín, Colombia; d Centro de Medicina del Ejercicio y Rehabilitación Cardíaca - CEMDE, Medellín, Colombia; e Universidad Nacional de Colombia - Sede Medellín, Facultad Ciencias, Departamento de Física, Grupo de Investigación de Física Radiológica, Carrera 65 No. 59A-110, Medellín, 050034, Colombia

**Keywords:** Body temperature, Exercise, Infrared thermography, Modified Bruce protocol, Temperature variation, Thermo Stress Evaluation

## Abstract

**Problem and motivation:**

The human body dissipates 60 % of its heat by emitting infrared radiation, it can be studied using Infrared Thermography (IRT). IRT images serve as thermal maps of the body, useful in medical applications to investigate the physiopathological of diseases that present symptoms such as swelling, pain, infection, rash, and increased local skin temperature.

**Aim:**

To design a protocol to capture IRT images before and after physical activity. The protocol collects skin temperature data of the entire body, in the frontal (anterior and posterior) and sagittal (right and left) planes.

**Methodology:**

The protocol was designed considering clinical, environmental, and technical factors and ensuring its reproducibility in both healthy and pathological populations. Thermographic images were acquired both at rest and after thermal stress (modified Bruce test). In addition, questionnaires were prepared to collect and store information on demographic data, core temperature, environmental conditions, pain perception, and level of physical activity.

**Results:**

The protocol combines the acquisition of IRT images with the application of the modified Bruce protocol on a treadmill as a thermal stress generator.

**Further impact:**

This protocol offers a valuable tool for studying the thermoregulatory capacity of the human body in the presence of different medical conditions.

## Graphical abstract

**Figure:**
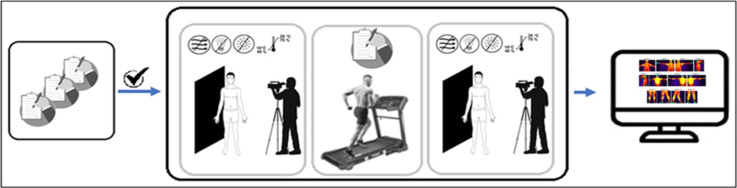

Specifications table

**Table:** 

Subject area:	Engineering
Method name:	Thermo Stress Evaluation
More specific subject area:	Biomedical Engineering
Name of your protocol:	*Protocol to evaluate human thermoregulation before and after thermal stress*
Reagents/tools:	The protocol does not require the use of equipment from specific manufacturers. However, the following minimum specifications should be met: • Thermal imaging camera with a minimum Focal plane arrays (FPA) of 320 × 240, a wavelength range between 8 and 12 µm, a temperature range between 20 °C and 120 °C, a maximum focal distance of 2.5 m, measurement traceability of 2 % of the overall reading and NETD (Noise Equivalent Temperature Difference) <30 mK. • Electric treadmill with variable speed and gradient settings • Portable pulse oximeter • Electronic thermometer with a minimum accuracy of ±0.1 °C • Digital thermo-hygrometer • Dark backdrop and platform • Computer (preferably a laptop)
Experimental design:	Cross-sectional experimental protocol to collect non-random intra-subject data
Trial registration:	N/A
Ethics:	Prior to their participation, all subjects were required to sign an informed consent form that had been previously approved by the Research Ethics Committee of the Instituto Tecnológico Metropolitano (Medellín, Colombia) on June 4, 2020.
Value of the Protocol:	• This protocol can be employed to study the thermoregulatory behavior of the human body by measuring skin temperature in real time using IRT and inducing thermal stress with a standardized exercise routine. • It is rapid, simple, non-contact, environmentally sustainable, innocuous, highly reproducible, and applicable to a wide range of subjects thanks to its integration of IRT and a modified Bruce test. • It is useful for investigating physiopathological conditions associated with various vascular phenomena, as well as any situation that results in alterations in skin temperature. This includes analyses of physical capacity; studies of joint, muscle, or tendon disorders; and follow-ups on infections, skin infections, swelling, pain, and inflammation, among others.

## Background

The human body radiates heat in the form of electromagnetic waves, which can be captured by thermal imaging cameras to create thermal profiles of specific regions of interest. The Bruce test is a standardized physical activity routine that can raise body temperature by increasing cardiovascular activity and engaging most muscle groups [1].

Infrared Thermography (IRT) can serve as a valuable supplementary tool in the diagnosis of various disorders associated with blood supply abnormalities. Additionally, it can assist in the understanding and treatment of some neurological diseases and pain syndromes. In some countries, this technology is also applied in the field of forensic medicine. Moreover, it is employed to assess pain mechanisms, as it identifies pathophysiological alterations within the internal environment that can explain pain [2].

Therefore, this study proposes a protocol that leverages IRT to evaluate human thermoregulation before and after thermal stress induced by the modified Bruce test. This protocol can be applied to athletes or individuals with painful pathologies, inflammatory conditions, cardiovascular and neurodegenerative diseases, and cancer, among others. During the development of this protocol, questionnaires were designed to collect demographic data, core temperature values, environmental conditions, pain perception, and physical activity levels. The resulting data can be stored in a database for the characterization of the participant group. Notably, to the best of our knowledge, this type of tool has not been employed in previous studies.

## Description of the protocol

The following environmental and technical parameters should be controlled to standardize the measurements in the protocol:

• **Environmental parameters**

To reduce variations in environmental conditions, the minimum measurement area should be 2 × 3 m. In addition, to prevent any influence on the body's thermal response, the ambient temperature should be maintained between 18 °C and 25 °C; and the relative humidity, between 40 % and 70 %. The IRT images should be captured with the lights off and the windows covered to minimize interferences from infrared frequencies emitted by natural or artificial light sources. Moreover, external air currents, heating pipes, and exposed electrical wiring should be avoided, and a dark backdrop should be installed to create a uniform background [3]. Placing a platform is also recommended for the participant to stand on so that the camera can be positioned perpendicular to the lower limbs.

• **Technical parameters**

The thermal imaging camera used should have a minimum Focal plane arrays (FPA) at least 320 × 240, a wavelength range between 8 and 12 µm, a temperature range of 20 °C to 120 °C, a maximum focal length of 2.5 m, measurement traceability of 2 % of the overall reading and NETD (Noise Equivalent Temperature Difference) <30 mK [3,4], and current calibration. The camera should be located at the same distance from the subject for all images and aligned parallel to the area of interest. Moreover, a tripod or stand is essential to keep the camera steady and to focus it at a 90° angle to the target [5].

The mean distance between the subject and the camera was calculated with Eq. (1), which determines the area (A) required to visualize images with the camera's field-of-view (FOV) which represent the vertical and horizontal aperture angles of the camera lens (``FOV'' = *β* × *α*) [6,7].

(1) $$d={\left[\frac{A}{4\;\times \tan\left(\frac{\beta }{2}\right)\times \tan\left(\frac{\alpha }{2}\right)}\right]}^{1/2}$$

For the physical activity section of the protocol, a treadmill with adjustable speed and inclination is required, preferably with front and side support for safety.

Core body temperature should be measured orally using an electronic thermometer with a minimum accuracy of ± 0.1 °C. Environmental conditions should be monitored with a digital thermohygrometer with an accuracy of ± 1 °C.

All equipment used in this protocol should undergo calibration routines according to the manufacturers’ recommendations.

## Steps of the protocol

• **Step 1**

Participants are selected based on inclusion and exclusion criteria, which should be defined according to the study population. Given that certain diseases or pathologies can alter body temperature, it is important that the study group has similar medical characteristics. Moreover, a control (healthy) group should be included for comparison. If a group of patients with a specific pathology is to be studied, the inclusion and exclusion criteria should be designed specifically for that pathology.

Participant characteristics are determined through questions about gender, age, weight, and height [8,9]. They are also asked if they have any recent lesions, scars, or tattoos, as these factors can cause slight variations in skin temperature [10]. Additionally, information is gathered on whether participants suffer from any diseases, experience pain, or have been diagnosed with a disability resulting from their condition, including their score on the Expanded Disability Status Scale (EDSS). This information is recorded using the Physiological Selection and Characterization (PSC) questionnaire (see supplementary material). Responses are stored in an Excel spreadsheet that contains conditional formulas for each question to quickly determine whether a subject qualifies for participation. In studies that implement this protocol, the PSC questionnaire can be adapted to apply specific inclusion and exclusion criteria.

Since this protocol involves physical activity to induce thermal stress, participants are classified based on their level of activity (Sedentary, Lightly Active, Moderately Active, or Active), as this could be a key factor in thermoregulatory behavior. Therefore, to assess physical activity, this protocol includes items from the Rapid Assessment of Physical Activity (RAPA) questionnaire [11], as well as questions about the duration, intensity, and flexibility of said activity (see supplementary material).

Participants should also follow additional recommendations, as certain factors may influence body temperature during image acquisition. First, they should not engage in intense physical activity twelve hours prior to the test and should not consume coffee, alcohol, energy drinks, or nicotine three hours before the test. Additionally, participants should not have applied creams, lotions, or oils at the time of testing to avoid alterations in skin emissivity [5,12,13]. The measurements should be taken in the morning for metabolic reasons, as core, distal, and proximal body temperatures tend to average at this time of the day [14].

• **Step 2**

IRT images are acquired in two stages: (S1) at rest and (S2) after physical activity.

Before S1 and S2, participants complete the Pain Perception Questionnaire (PPQ; see supplementary material) to assess the pain they experience in different body areas. Specifically, the PPQ features four illustrations of a human body (posterior, anterior, left, and right), with text boxes in each body part for participants to rate their pain on a scale from 1 to 10. There are also description boxes for researchers to add any relevant comments on the pain reported by participants. The PPQ also records core body temperature, ambient temperature, and relative humidity values (see Fig. 8). Once the answers are submitted, the information is stored in an Excel spreadsheet and added to a database, which will be used to identify the body areas where pain is felt before and/or after physical activity, as well as the intensity of that pain.

**Fig. 1:**
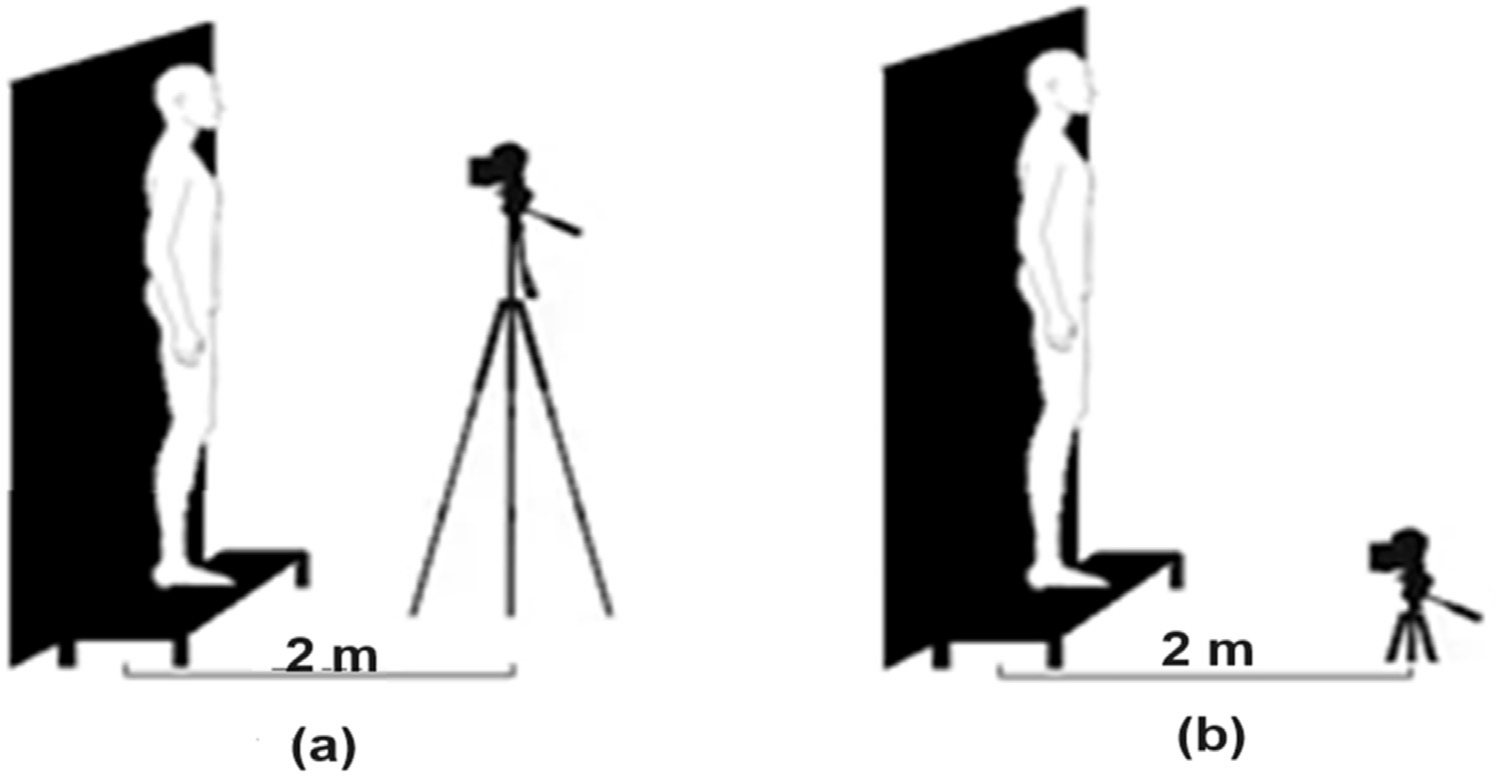
Thermal imaging camera positions for image acquisition.

The thermal imaging camera should be securely mounted on a tripod, leveled, and positioned approximately 2 m from the target. The camera's height may be adjusted as needed, always ensuring that it remains perpendicular to the body regions of interest (see Fig. 1). It is advisable to use markers to position both the participants and the camera to maintain the recommended distance during the measurements. In some cameras, the skin emissivity value must be set directly on the device; in others, it can be entered during image processing. In both cases, the reference value is 0.98 [3,15].

**Fig. 2:**
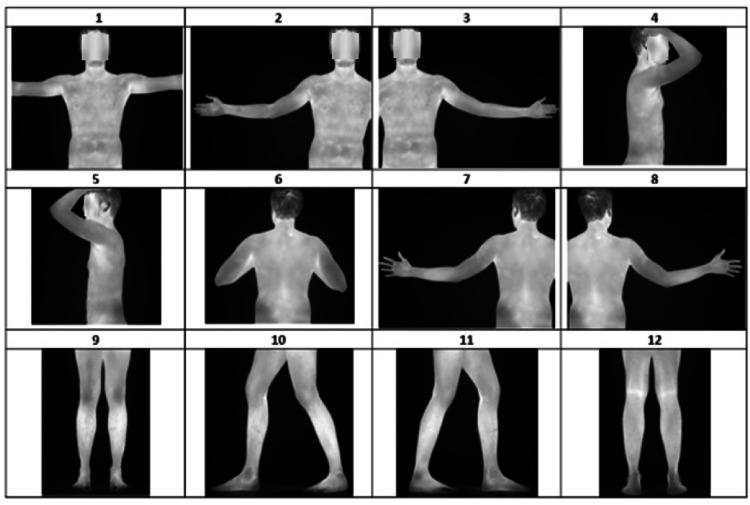
Positions for acquiring IRT images before (S1) and after (S2) physical activity.

For S1, participants must remain at rest for 10 to 15 min to acclimate and allow their baseline thermal profiles to stabilize. After this period, they must stand on the platform, in front of the dark backdrop, and adopt the positions shown in Fig. 2.

The upper-body images capture the area from the forehead to the iliac crests, while the lower-body images cover the region from the inguinal groove to the soles of the feet. The details of each position are listed in Table 1.

**Table 1:** Positions for capturing full-body thermograms.

Position	Plane	Lateral limits	Position
**1**	Anterior	Elbow creases	Shoulders abducted to 90°
**2**		Entire right arm up to the left shoulder	
**3**		Entire left arm up to the right shoulder	
**4**	Right	Elbow and back	Right hand on the head and arm flexed at 90°; left hand in anatomical position
**5**	Left		Left hand on the head and arm flexed at 90°; right hand in anatomical position
**6**	Posterior	Elbows	Shoulders abducted to 45°, with arms flexed
**7**		Entire left arm up to the right shoulder	Shoulders abducted to 90°
**8**		Entire right arm up to the left shoulder	
**9**	Anterior	Hip width	Standing with feet shoulder-width apart
**10**	Left	Toe of the right foot and heel of the left foot	Right foot in front of left foot
**11**	Right	Toe of the left foot and heel of the right foot	Left foot in front of right foot
**12**	Posterior	Hip width	Standing with feet shoulder-width apart

Once the images have been acquired in S1, the participant's baseline Heart Rate (HR) should be measured. Subsequently, the participant must stand on the treadmill to start the modified Bruce protocol, following the sequence outlined in Table 2. It is important to explain the test procedure to each participant, emphasizing that the stages are continuous, with each stage lasting three minutes, and that, as the test progresses, the activity becomes increasingly challenging. The activity concludes when the participant decides to stop or when the researcher deems it necessary, either because there is an imminent risk of falling (due to exhaustion or fainting) or because of an excessive increase in HR.

**Table 2:** Sequence of the modified Bruce protocol.

Stage	Time [min]	Speed [Km/h]	Gradient [%]	Approximate METs
**1**	3	2.7	0	1.7
**2**	3	2.7	5	2.8
**3**	3	2.7	10	5.4
**4**	3	4.0	12	7.0
**5**	3	5.4	14	10
**6**	3	6.7	16	13
**7**	3	8.0	18	17
**8**	3	8.9	20	20

At this point, the researcher uses the Physical Activity Response (PAR) questionnaire (see supplementary material) after each stage of the modified Bruce protocol to record the HR and perceived exertion according to the Borg scale presented in Table 3.

**Table 3:** Borg scale.

**0**	Total rest
**1**	Very light
**2**	Light
**3**	Moderate
**4**	Somewhat hard
**5**	Hard
**6**	
**7**	Very hard
**8**	
**9**	
**10**	Maximal

These data will be used to evaluate additional factors, such as the perceived exertion of each participant and the individual cardiovascular endurance achieved during physical activity. This evaluation will be based on the theoretical Maximum Heart Rate (MHR), which is calculated using Eq. (2).

(2) TheoreticalMHR=208.75−(0.73×age)

After the physical activity is completed, the PPQ is filled out again. Following this, the images for S2 are acquired, using the same procedure as in S1.

**Fig. 3:**
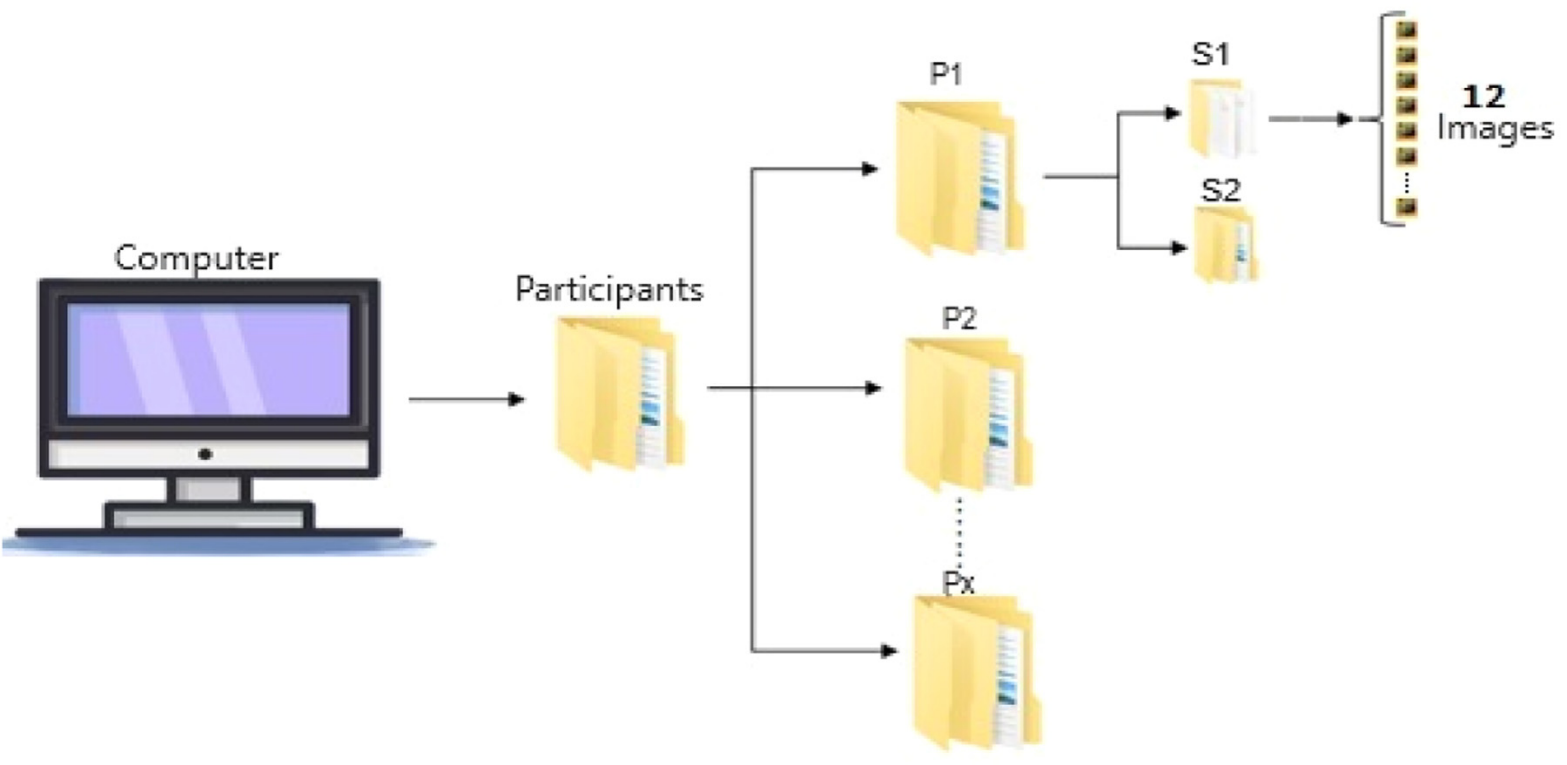
Folder structure for storing the images.

The images should be stored in an orderly manner, sorted by participant and stage. To facilitate this process, each participant is assigned a code that starts with the letter P followed by a number (e.g., the first participant is assigned P1; the second participant, P2; and so forth). A folder is created for each participant, named according to their assigned code. In turn, each folder contains two subfolders named S1 and S2 to differentiate between the images obtained before and after physical activity (see Fig. 3).

The images can be converted into formats such as MAT, CSV, or matrices compatible with MATLAB.

• **Step 3**

The thermograms acquired in S1 and S2 are processed to find the average temperature of the body areas captured in each image.

To process the images in MATLAB, the thermograms should be saved in MAT file format using the structure shown in Fig. 3. The algorithm reads the images; removes background noise, retaining only the pixel information associated with the participant's body; and then applies first-order statistical texture analysis. This analysis calculates up to six texture measurements, including the average temperature, which is derived from the grey levels of the body surface pixels in each IRT image. Therefore, the data processing yields twelve average temperatures, corresponding to the twelve images of each participant and stage.

## Protocol validation

The protocol was applied to two subjects with different medical profiles. The first was a healthy individual without any medical conditions (i.e., the control subject). The second subject was a patient diagnosed with Multiple Sclerosis (MS). The objective was to study differences in the skin's thermoregulatory behavior after exposure to thermal stress.

## Equipment and software

The images were acquired using a FLIR A655sc thermal imaging camera (FLIR Systems Inc., Oregon, US) owned by the Instituto Tecnológico Metropolitano (ITM) in Medellín, Colombia. This camera has a FPA of 640 × 480, a spectral range of 7.5 to 14 µm, measurement traceability of 2 % of the overall reading and NETD (Noise Equivalent Temperature Difference) <30 mK. Each pixel in the images indicates the local temperature of the corresponding area.

The physical activity used to induce thermal stress (i.e., increase body temperature) was performed on a Sportop PRO700 treadmill owned by the ITM's Biomechanics and Clinical Rehabilitation Laboratory.

During the physical activity, participants’ HR was measured using a CoreRay CR-50 portable pulse oximeter (Shenzhen Core Ray Technology Co., Ltd) with a pulse rate range of 30 to 250 bpm, a resolution of 1 bpm, and an accuracy of ±2 bpm or 2 %.

The core body temperature was measured with a Welch Allyn SureTemp® Plus 690 electronic thermometer (Welch Allyn®, US) set for oral measurements. This device displays the core body temperature within six seconds and can measure temperatures ranging from 26.7 °C to 43.3 °C, with an accuracy of ±0.1 °C. Moreover, environmental conditions, such as ambient temperature and relative humidity, were measured using a digital thermo-hygrometer.

A dark backdrop was placed behind the participants to have a more uniform background and improve thermogram quality. Furthermore, when acquiring lower-body images, a platform was employed to ensure the perpendicularity of the camera.

FLIR ResearchIR software, version 4.4, (FLIR Systems Inc., Oregon, US) was used to capture, edit, and import the IRT images. Additionally, the algorithm presented in this paper was employed in MATLAB, version 2021a, (MathWorks Inc., Massachusetts, US) for image processing. All these tasks were performed on an HP 14-ck0013la laptop.

## Measurement environment

The measurements were performed in the Biomechanics and Physical Rehabilitation Laboratory at ITM in Medellín, Colombia. The indoor space, measuring 35 m^2^, is equipped with air conditioning to maintain controlled environmental conditions. During the study, the ambient temperature was kept between 19 °C and 23.3 °C; the relative humidity, between 40 % and 64 %.

To ensure that participants had similar characteristics, the following inclusion and exclusion criteria were applied (Table 4).

• **Step 1**

**Table 4:** Inclusion and exclusion criteria.

Criterion		Control	MS
**Inclusion**	Age	18–65	
	Confirmed diagnosis	NA	Yes
	Score on the EDSS	NA	< 6.5
	Signed informed consent	Yes	Yes
**Exclusion**	Difficulty understanding instructions or answering questions	Significant cognitive impairment; medical or psychological disorder	
	Presence of any disease	Cardiac or respiratory conditions, severe depression, fibromyalgia, severe arthritis, neurological disorders, and any other conditions considered by the researchers during the interview	
	Presence of any life-threatening condition	Any comorbidity that prevents participation in physical exercise	
	Excluded from the study	Medical recommendation, pregnancy, other pathologies	

## Participant selection and characterization

**Fig. 4:**
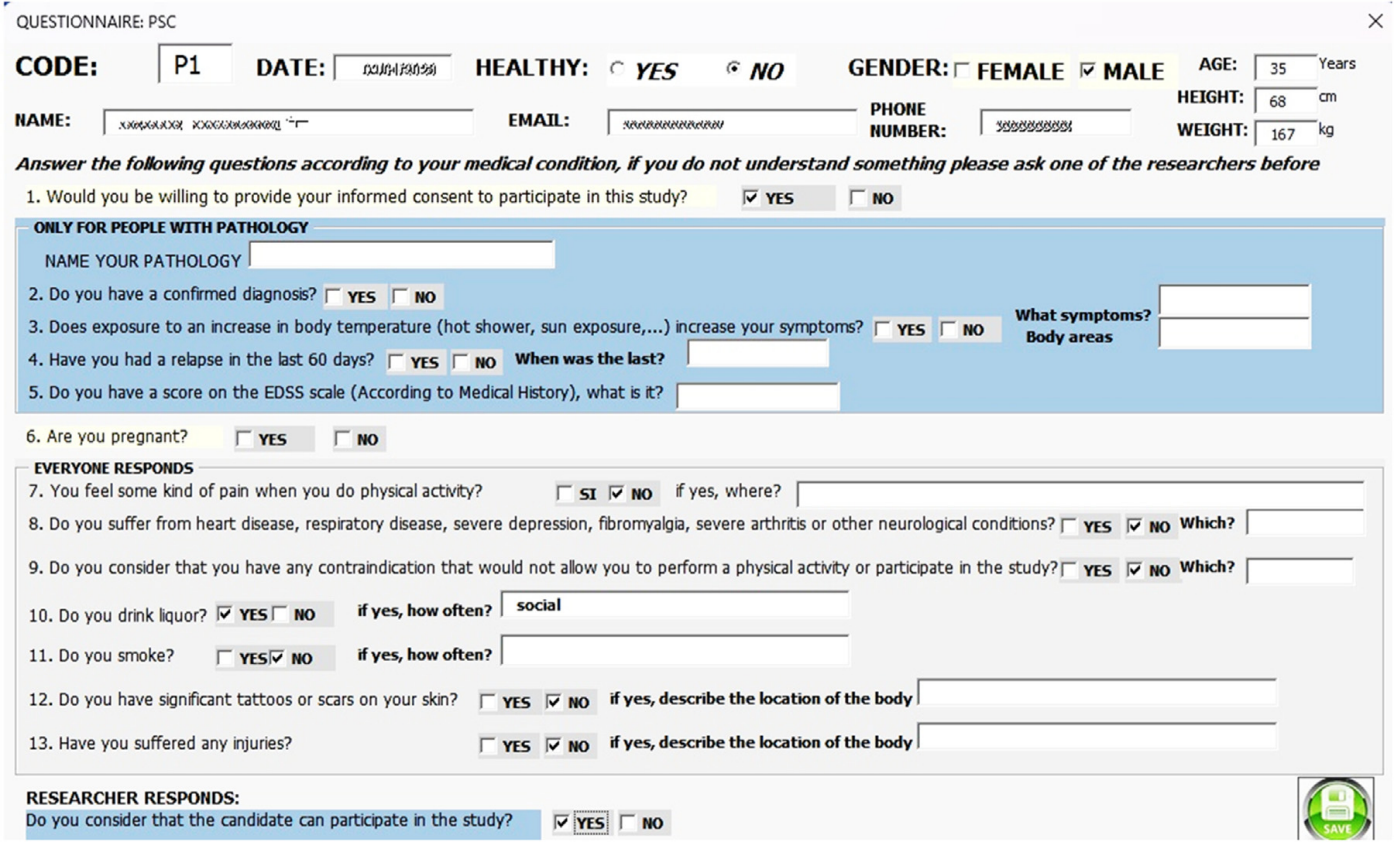
Interface for completing the PSC questionnaire.

First, participants completed the PSC questionnaire, which served to both characterize them and apply the inclusion and exclusion criteria described above (see Fig. 4).

**Fig. 5:**
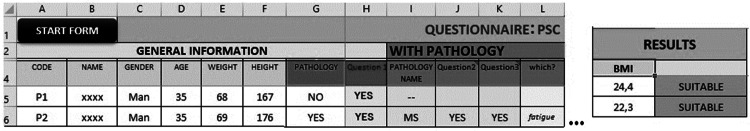
Results of the PSC questionnaire.

After completing the questionnaire, participants were provided with general information about the study and the recommendations detailed in Step 1 of the protocol (Fig. 5).

**Fig. 6:**
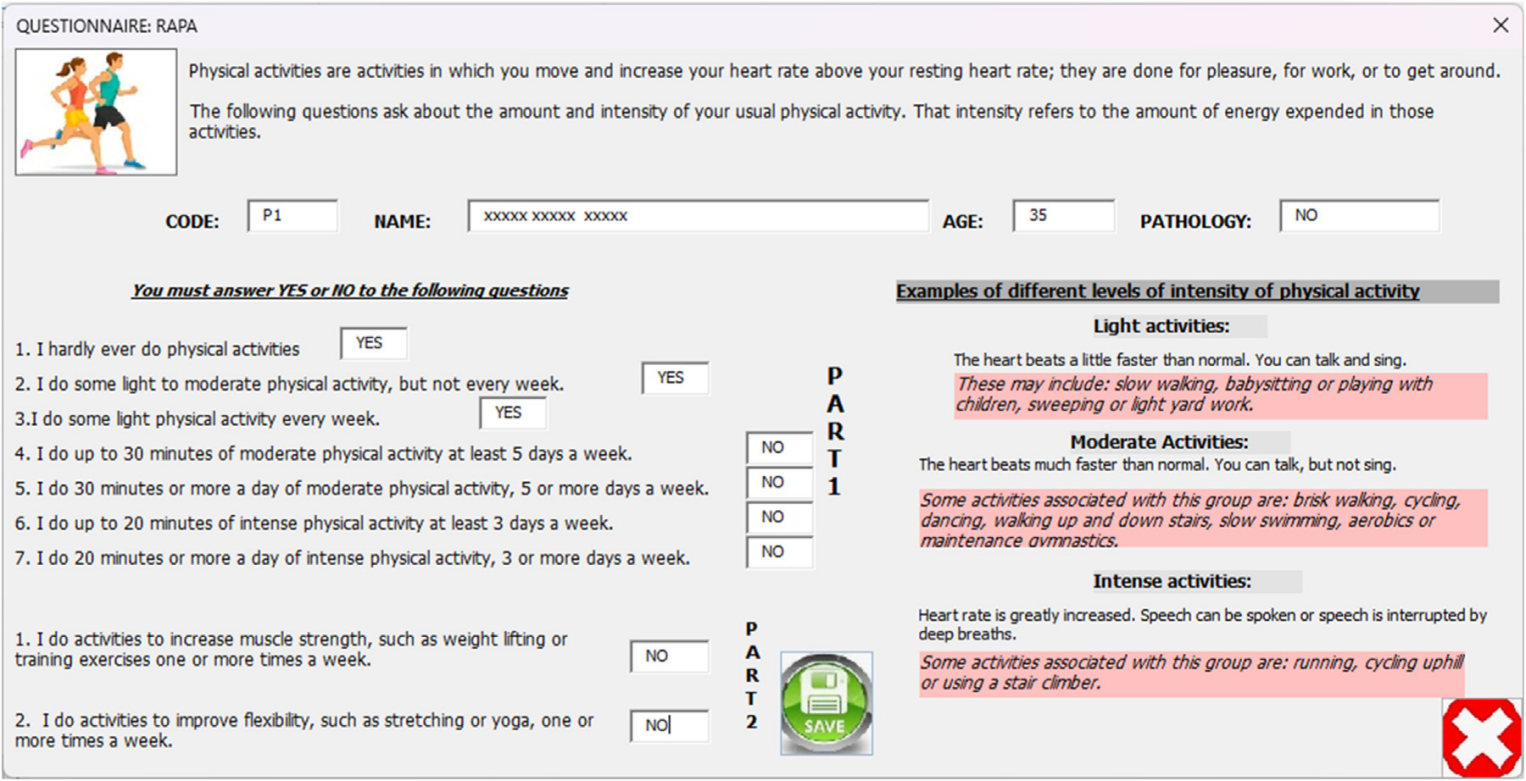
Interface for completing the RAPA questionnaire.

**Fig. 7:**
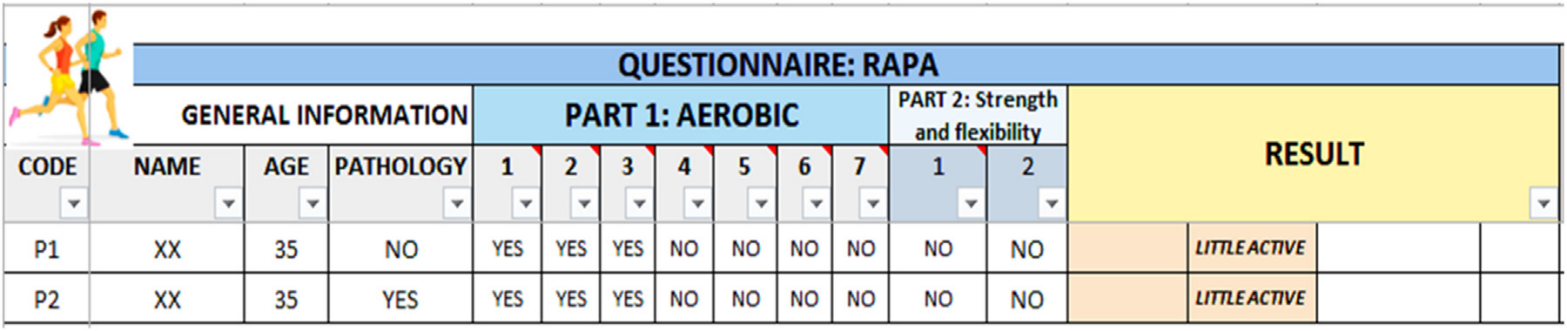
Results of the RAPA questionnaire.

Next, participants filled out the RAPA questionnaire (Fig. 6) to categorize their activity level as Sedentary, Lightly Active, Moderately Active, or Active (Fig. 7).

• **Step 2**

## Image acquisition

**Fig. 8:**
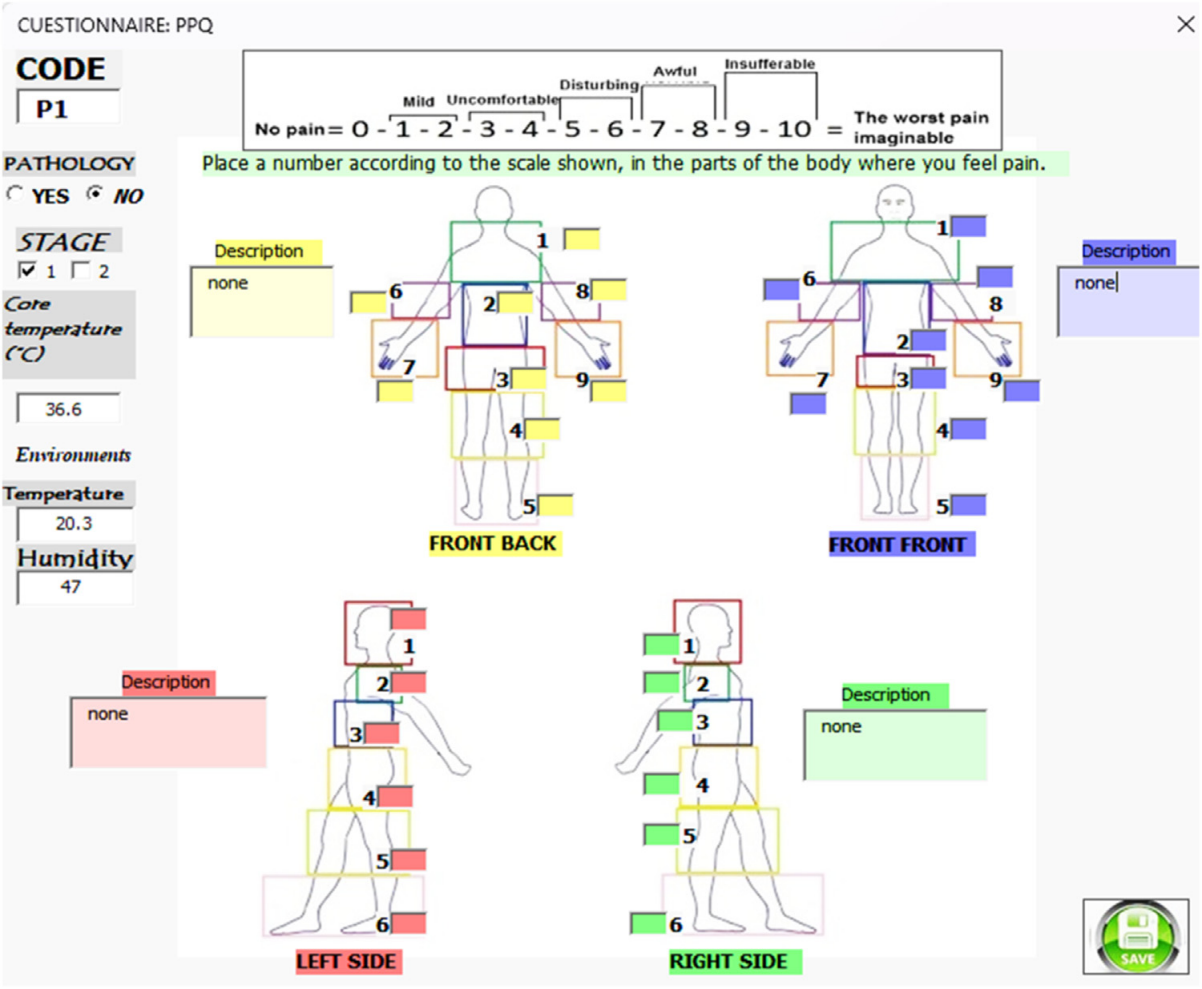
Interface for completing the PPQ questionnaire.

Subsequently, participants completed the PPQ questionnaire (Fig. 8) to record their pain levels.

**Fig. 9:**
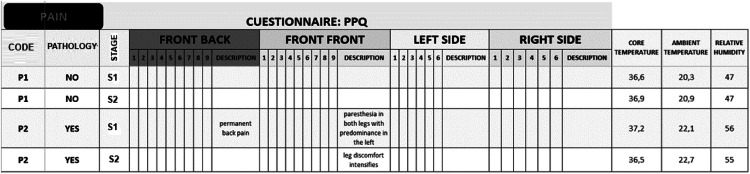
Results of the PPQ questionnaire.

During S1, the participant with MS reported paresthesia in both legs, predominantly in the left leg, as well as persistent back pain (see Fig. 9).

**Fig. 10:**
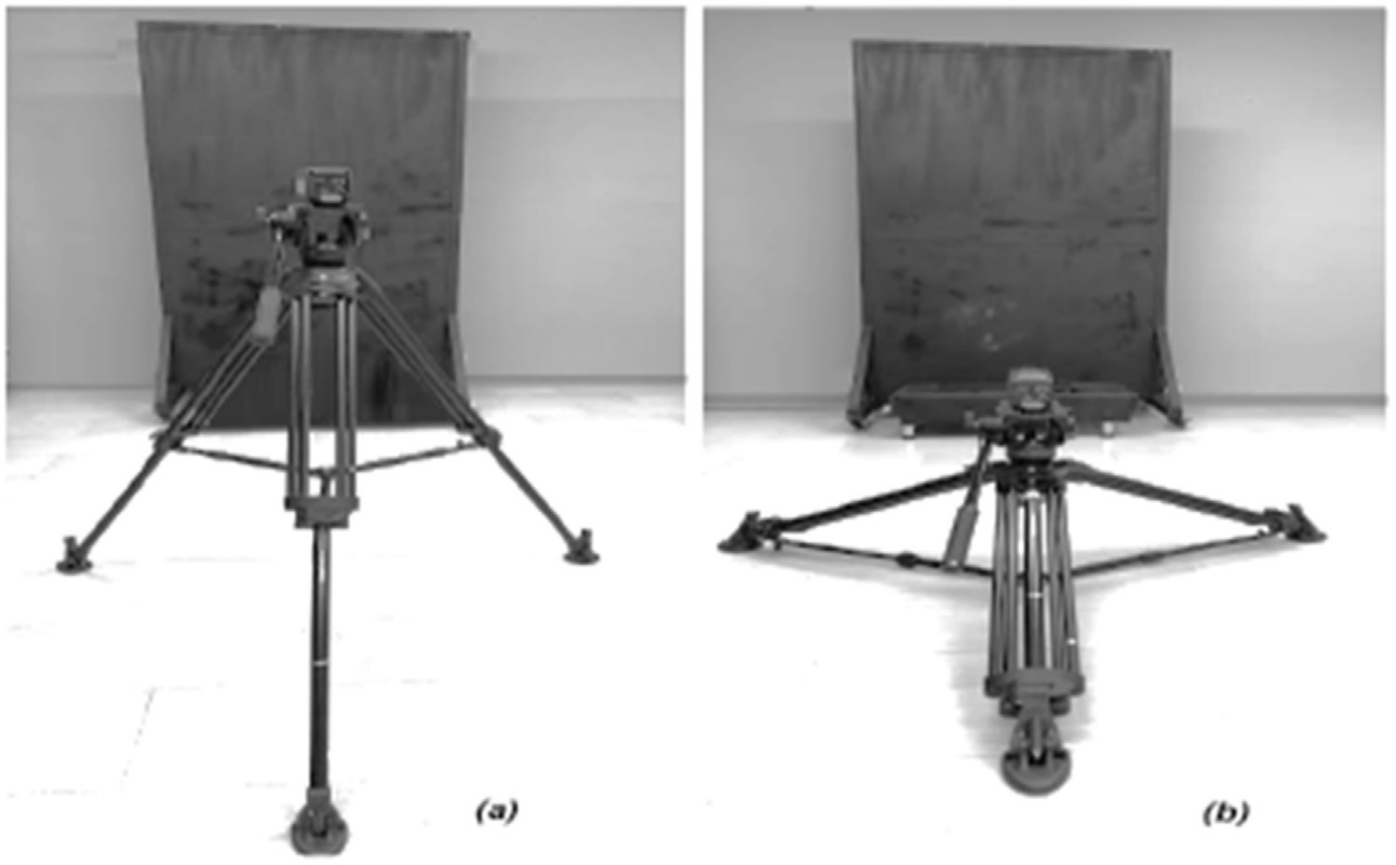
Camera positioning for (a) upper-body and (b) lower-body image acquisition.

The recommendations in the protocol were followed during image acquisition, with participants positioned on markers to ensure a consistent distance of 2 ± 0.2 m between the subject and the camera lens (see Fig. 10).

After capturing the S1 images, participants moved to the treadmill, where they received instructions on its use and an explanation of the steps of the protocol.

**Fig. 11:**
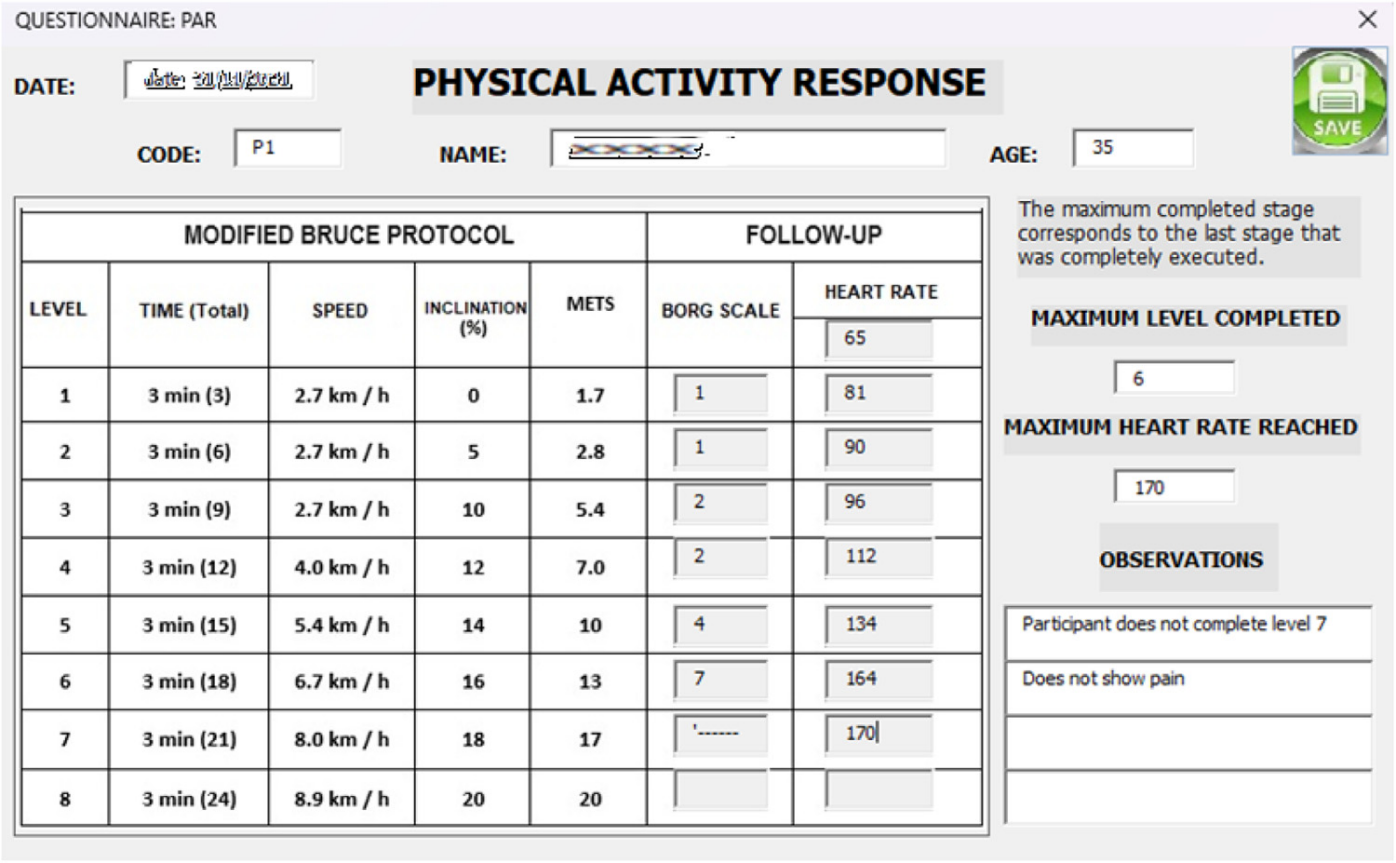
Interface for completing the PAR questionnaire.

During each stage of the modified Bruce test, the researcher measured participants’ HR and perceived exertion, which were then recorded in the PAR questionnaire (see Fig. 11).

**Fig. 12:**
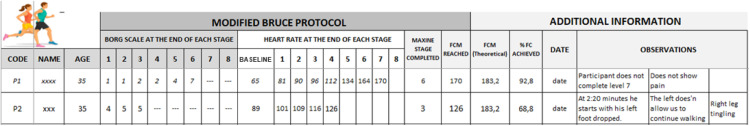
Results of the PAR questionnaire.

The data collected in the PAR questionnaire were subsequently exported to an Excel spreadsheet, where the theoretical MHR was calculated and used to determine the percentage of cardiovascular endurance achieved by each participant during the physical activity (see Fig. 12).

The physical activity concluded when each participant decided to stop. Throughout this activity, the researcher made sure that participants did not exhibit any symptoms, such as dizziness, chest pain, shortness of breath, or exhaustion. During the protocol validation, no participant required to stop the activity.

Immediately after the exercise, the S2 IRT images were captured using the parameters outlined above, and the PPQ questionnaire was filled out once more.

• **Step 3**

## Image processing

**Fig. 13:**
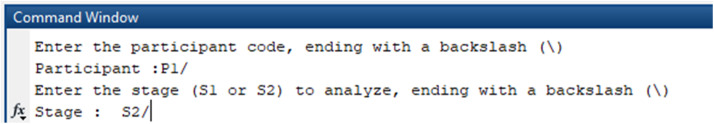
Data to be entered when executing the algorithm.

ResearchIR software was used to export the IRT images to MAT format, which is compatible with MATLAB. These files were then stored and processed as shown in Fig. 13.

**Fig. 14:**
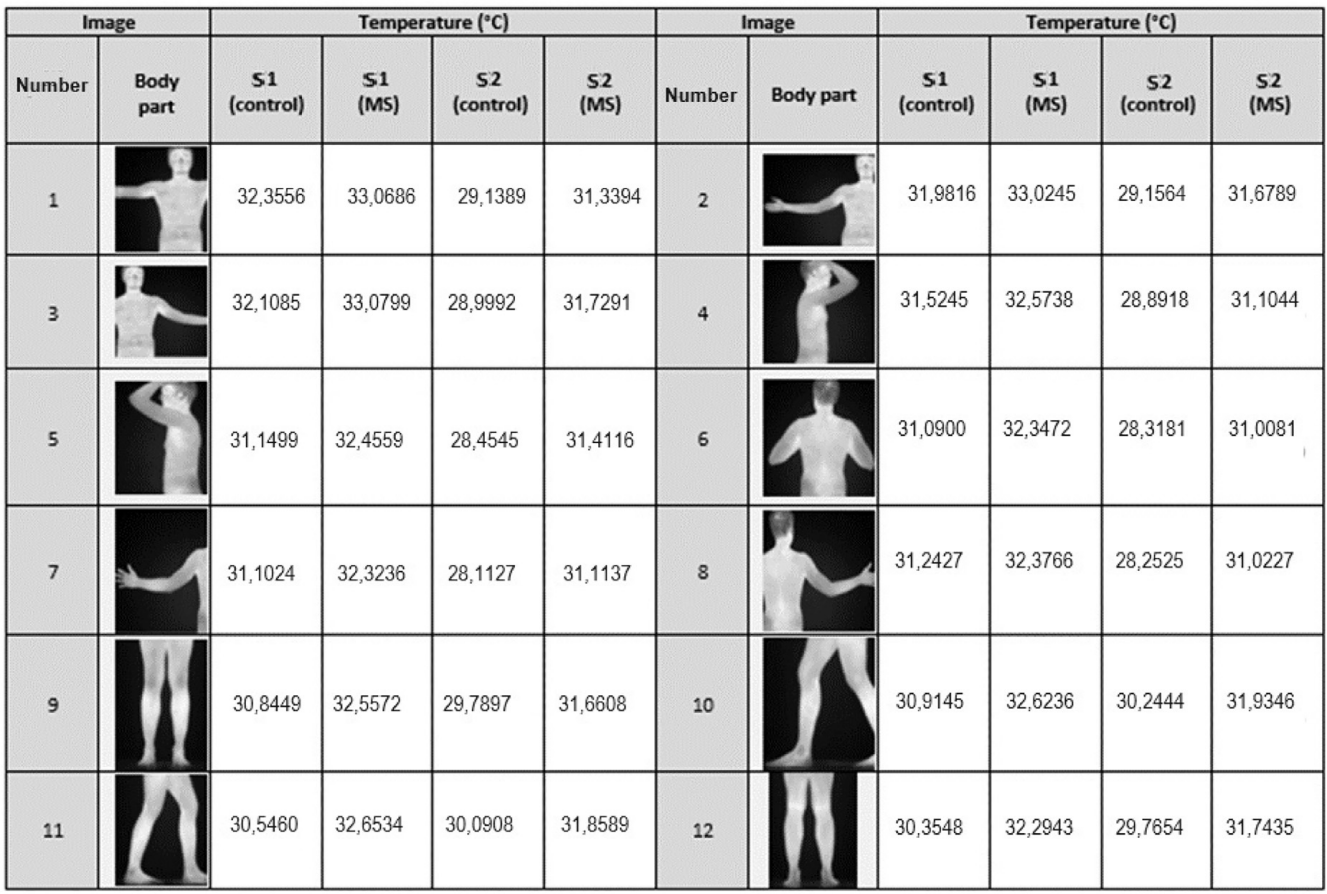
S1 and S2 temperatures for each participant.

When the MATLAB algorithm is executed, the user must enter the participant's code and the stage to be analyzed. The algorithm generates an Excel file containing twelve temperature values that correspond to the twelve images of that participant at that stage. The data are presented in the same order as the saved images. The algorithm automatically names and stores the file according to the preset specifications (see Fig. 14).

**Figure:**
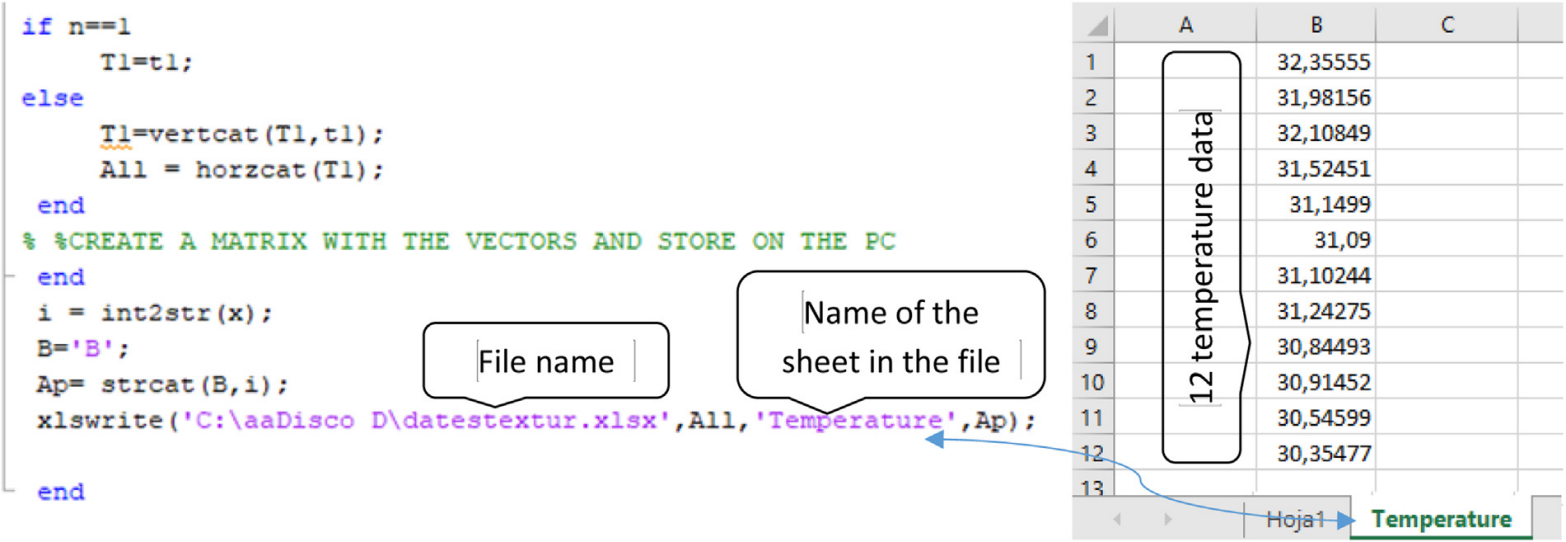
Creation of the Excel data file.

## Analysis of the data from the protocol

Fig. 14 presents the temperatures obtained after processing each IRT image, detailing the image number, body part, and average temperatures for each participant in both S1 and S2.

**Fig. 15:**
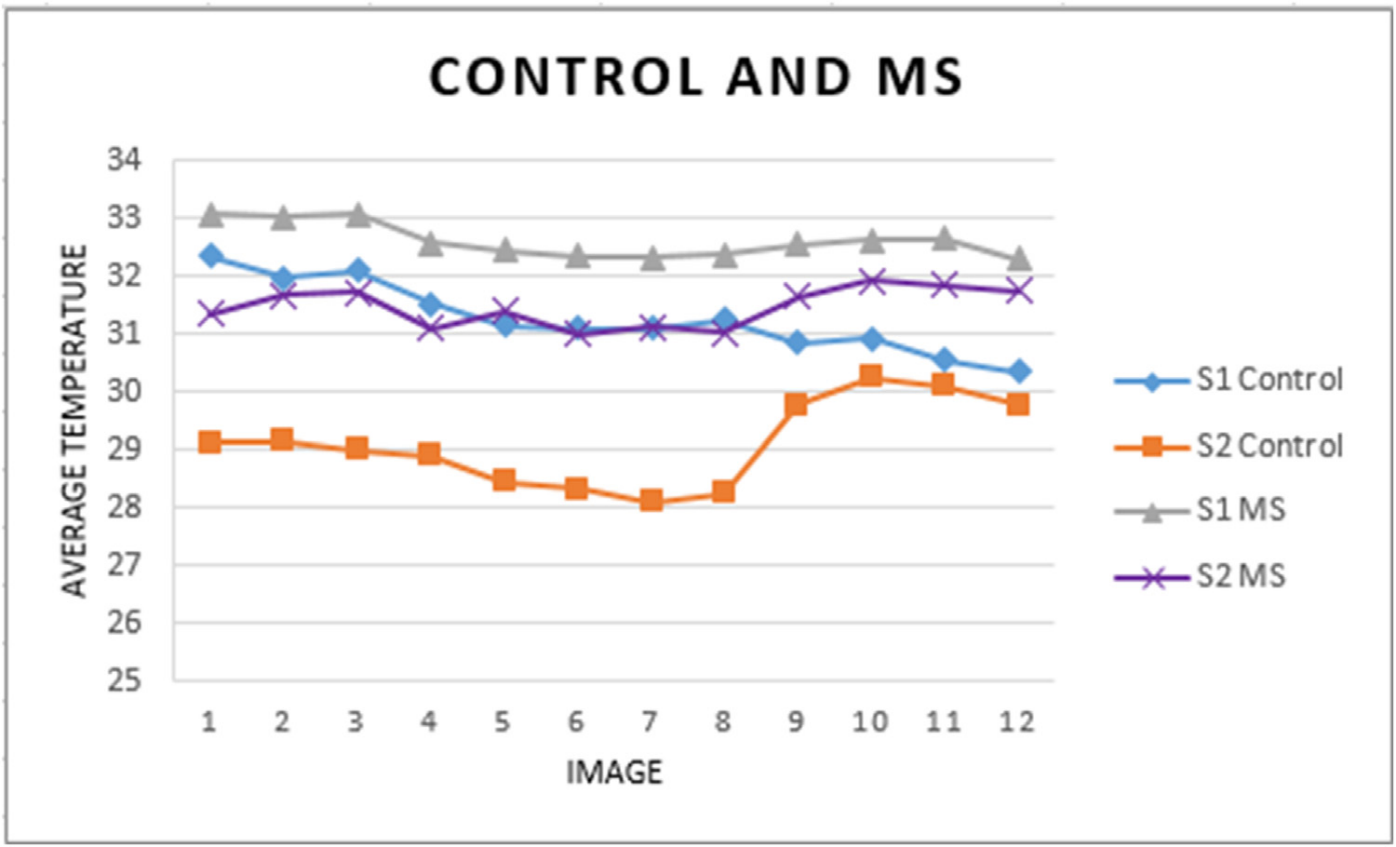
S1 and S2 temperature behavior for MS and control participants.

Fig. 15 shows the changes in average temperature (10 images) for MS and control participants before (S1) and after (S2) the physical activity.

**Fig. 16:**
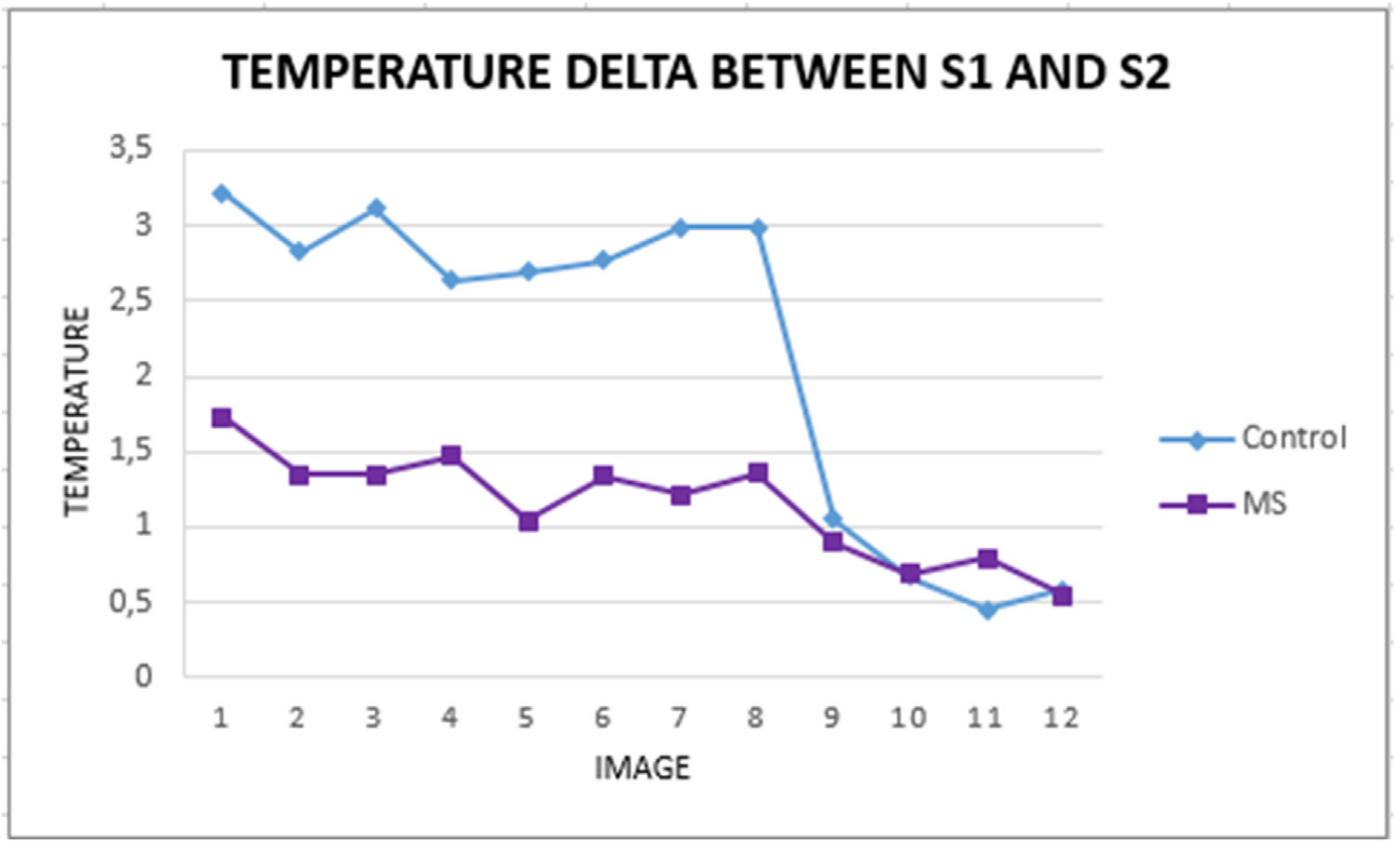
Temperature variation between S1 and S2 for each participant.

To identify variations, Fig. 16 presents the S1 and S2 temperature delta between each pair of images of the two participants.

In Fig. 15, the participant with MS (line with triangles: ▲) exhibited higher temperatures at S1 and S2 than the control participant. Additionally, after the physical activity, both participants experienced a decrease in body temperature, with the control participant showing a more significant drop in the upper body. The smallest temperature variation occurred in the lower body for both participants.

These findings suggest a difference in body temperature behavior between the participants, even though they had similar physical characteristics, such as age, sex, and BMI, and both being classified as Lightly Active. The biggest difference between them is that one participant has MS. This result indicates that this protocol can detect differences in body temperature behavior between healthy individuals and those with neurological, endocrine, or metabolic disorders. Although these variations are subtle and hard to detect, future studies could further explore this hypothesis with larger samples.

## Limitations

Participants should not have a disability that prevents them from walking on a treadmill. Moreover, the measurement environmental conditions should be controlled.

## CRediT authorship contribution statement

**Mariluz Castrillón-Gutiérrez:** Conceptualization, Data curation, Writing – original draft, Writing – review & editing. **Natali Olaya-Mira:** Conceptualization, Writing – original draft, Writing – review & editing. **Carolina Viloria-Barragán:** Conceptualization, Writing – review & editing. **Julieta Henao-Pérez:** Conceptualization, Methodology. **Edison Alejandro Álvarez -David:** Conceptualization, Methodology. **Gloria Díaz-Londoño:** Software, Writing – original draft, Writing – review & editing, Conceptualization, Supervision, Validation.

## Declaration of competing interest

The authors declare that they have no known competing financial interests or personal relationships that could have appeared to influence the work reported in this paper.

## Data Availability

Data will be made available on request.

## Appendix Supplementary materials

Image, application 1

Image, application 2

Image, application 3

Image, application 4
